# A Stochastic Simulator of a Blood Product Donation Environment with Demand Spikes and Supply Shocks

**DOI:** 10.1371/journal.pone.0021752

**Published:** 2011-07-26

**Authors:** Ming-Wen An, Nicholas G. Reich, Stephen O. Crawford, Ron Brookmeyer, Thomas A. Louis, Kenrad E. Nelson

**Affiliations:** 1 Department of Mathematics, Vassar College, Poughkeepsie, New York, United States of America; 2 Department of Biostatistics, Johns Hopkins Bloomberg School of Public Health, Baltimore, Maryland, United States of America; 3 Department of Epidemiology, Johns Hopkins Bloomberg School of Public Health, Baltimore, Maryland, United States of America; 4 Department of Biostatistics, University of California Los Angeles, Los Angeles, California, United States of America; Genentech Inc., United States of America

## Abstract

The availability of an adequate blood supply is a critical public health need. An influenza epidemic or another crisis affecting population mobility could create a critical donor shortage, which could profoundly impact blood availability. We developed a simulation model for the blood supply environment in the United States to assess the likely impact on blood availability of factors such as an epidemic. We developed a simulator of a multi-state model with transitions among states. Weekly numbers of blood units donated and needed were generated by negative binomial stochastic processes. The simulator allows exploration of the blood system under certain conditions of supply and demand rates, and can be used for planning purposes to prepare for sudden changes in the public's health. The simulator incorporates three donor groups (first-time, sporadic, and regular), immigration and emigration, deferral period, and adjustment factors for recruitment. We illustrate possible uses of the simulator by specifying input values for an 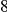-week flu epidemic, resulting in a moderate supply shock and demand spike (for example, from postponed elective surgeries), and different recruitment strategies. The input values are based in part on data from a regional blood center of the American Red Cross during 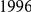
–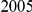. Our results from these scenarios suggest that the key to alleviating deficit effects of a system shock may be appropriate *timing* and *duration* of recruitment efforts, in turn depending critically on anticipating shocks and rapidly implementing recruitment efforts.

## Introduction

The availability of an adequate supply of blood and blood components for transfusion is a critical public health need. A national survey of blood collection centers and hospitals in the United States in 2001 estimated that 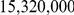 units were available, prior to screening [1]. This was 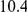 per cent greater than in 1999 [2]. However, transfusion of whole blood and red blood cells (RBCs) increased by 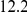 percent during this period. Although the rate of blood collection per 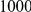 eligible donors in 2001 was 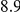 percent higher than in 1999, some of this increase was due to a 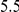-fold increase in first-time donors and a 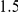-fold increase in repeat donors in response to the September 11, 2001 attacks on the World Trade Center in New York. However, despite this increase in the population-based rate of donation, the utilization of blood and blood products increased by 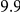 percent during this period.

In addition to these overall trends in blood collection and utilization, there are regional and seasonal trends that have important effects on the availability and need for blood and blood products. For example, blood collections regularly decrease during the summer months, especially near the July 4 and Labor Day holidays, and again during the Christmas holiday period [3]. Because the increased utilization of blood has surpassed the increased collection and because of the aging of the population, it is likely that periodic shortages in available blood will be more frequent in the future if the current donation trends continue.

Another concern is that a severe epidemic of influenza or another crisis affecting population mobility could create a critical shortage of donors, which could have a more profound and longer lasting impact on blood availability.

In order to better understand the variability of the blood supply, the risks of a critical shortage of blood and blood components, and the ability of the system to adjust to circumstances of changes in availability and demand, we developed a simulation model. This simulation model captures the variation in supply and demand in a closed system, and can be used for planning by varying the input parameters. The model considers three types of donors (first-time, sporadic, and regular) and non-donors in the population and the effect that quantitative changes in their availability could have on the supply of blood and components. For simplicity, this model is set to simulate the overall supply and demand of red blood cells or whole blood, without considering different blood types of red blood cells or other blood labile components. The primary goal of our paper is to introduce the simulator as a freely available tool for others to use and enhance. Further, to illustrate one possible use of the simulator, we include an example motivated by a possible flu epidemic wherein we specify parameter values based, in part, upon data from a regional blood center of the American Red Cross during the time period from 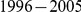.

This paper is organized as follows. First, we describe the structure of the simulator and its inputs in general form, introduce notation, and state assumptions; then we illustrate one example use of the simulator by specifying parameter values for two principal scenarios: “normal conditions” and “moderate supply shock and demand spike,” and presenting results from sample runs representing different recruitment strategies in response to the shock and spike; and we conclude with a discussion and identify future work. The simulator was coded in the statistical software R version 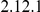 [4], and the code is available upon request.

## Methods

### 3.1 Multi-state, Stochastic Model

**Figure 1:**
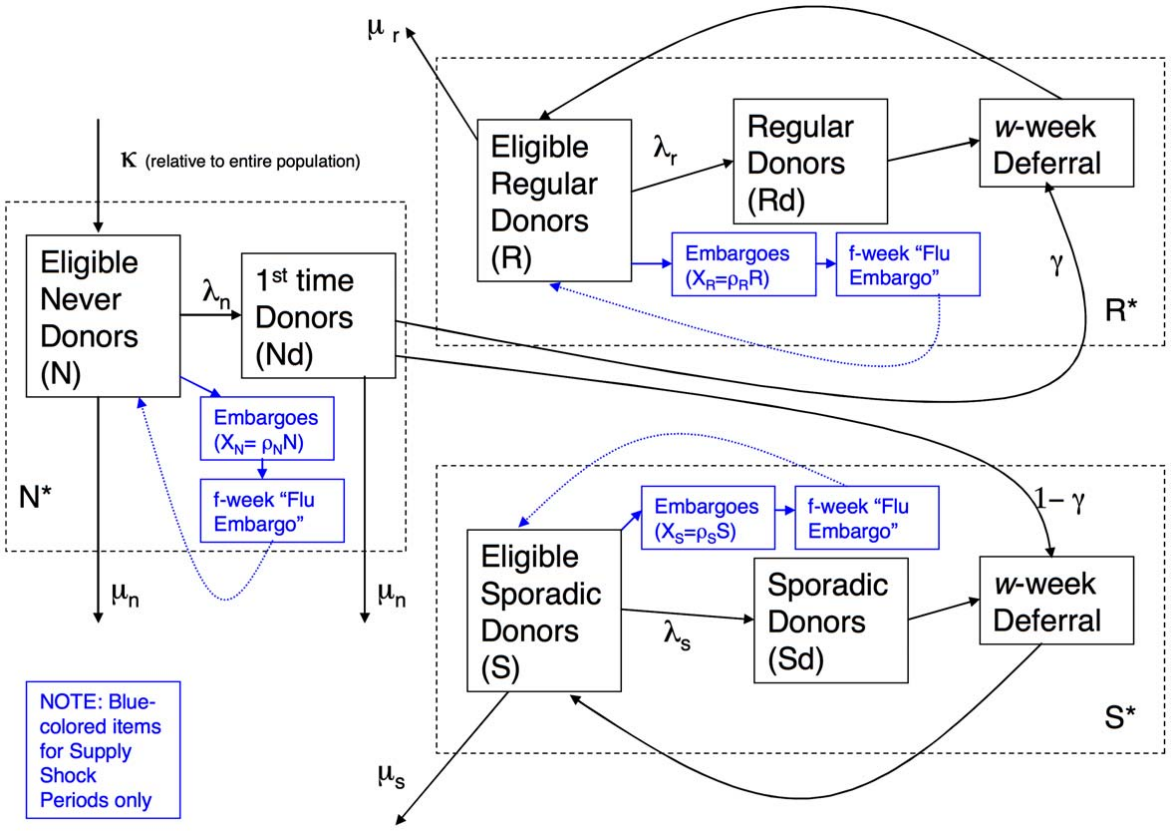
Schematic of the Blood Donation System with three donor states, immigration and emigration, donation and deferral, and transition from first-time donor into either Regular or Sporadic states.

The simulator is based on a multi-state model with transitions among three states (never, sporadic, and regular donors), the possibility of a flu epidemic or other event limiting blood supply (a supply shock), and the possibility of an event producing an abrupt increase in the need for blood products (a demand spike). In this section, we describe the key features of the simulator. In the next section, we provide more details with notation. We also refer the reader to Figure 1, which displays a schematic of the simulator; as well as to the first two columns of Tables 1 and 2, which summarize the parameters described in this section. Full details are available in the Appendix S1.

**Table 1:** Input Parameters for a “Normal” Period (informed by expert opinion or data).

Description	Notation	Example Values
No. of simulated weeks		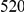
Baseline donor population sizes		
Total	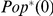	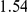 M
Regular	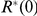	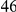 K
Sporadic	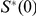	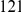 K
Never	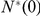	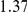 M
Standard deferral period following any donation (weeks)	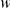	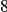
Shelf-life of a donated unit (weeks)	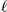	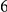
Weekly donation rate for regular donors	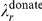	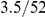
Weekly donation rate for sporadic donors	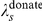	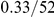
Weekly first-time donation rate for never-donors	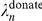	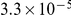
Immigration rate into donor system, relative to size of total population	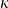	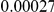
Emmigration rates		
from Regular donor pool	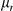	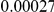
from Sporadic donor pool	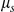	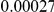
from Never-donor pool	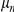	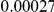
Regular programmatic adjustments to recruitment		
maximum available supply threshold	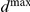	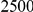
minimum available supply threshold	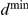	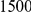
adjustment factor to increase donation	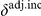	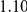
adjustment factor to decrease donation	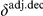	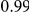
Fraction of non-emigrating first-time donors who become regular	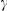	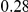
(vs. sporadic) donors		
Overdispersion rates		
for Regular donor rate	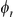	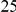
for Sporadic donor rate	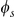	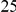
for First-time donor rate	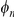	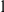
for weekly demand rate	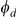	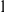
Usual weekly demand rate	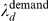	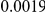

**Table 2:** Additional Parameters for a Hypothetical Period with Supply Shock, Demand Spike, and Enhanced Recruitment.

Description	Noatation	Example Values
Supply		
Proportion of regular donors ineligible due to flu	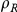	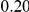
Proportion of sporadic donors ineligible due to flu	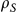	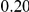
Proportion of never-donors ineligible due to flu	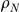	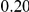
Flu embargo period (weeks)	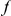	4
Flu epidemic interval	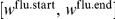	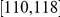
Demand		
Multiplicative factor applied to usual demand rates	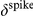	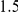
Demand spike interval	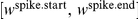	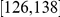
Enhanced Recruitment		
Multiplicative factor applied to usual donation rate	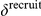	*
Recruitment effort interval	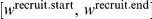	*

*: Refer to Table 3 for specific scenarios.

#### 3.1.1 The States

At the beginning of week 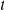, each individual in a reference population is in one of three donor states: “regular,” “sporadic,” or “never.” Anyone who has ever donated is in either the regular or the sporadic state with regular donors having a higher donation rate than the sporadic. The “never” state includes individuals in the reference population who have yet to donate. They become first-time donors at a specified rate. First-time donors then emigrate from the system, become regular donors, or become sporadic donors at specified rates. After donating, an individual enters a deferral period of a specified number of weeks during which donation is not allowed. Donors remain either regular or sporadic, or emigrate from the system.

#### 3.1.2 Supply and Demand

*Stochastic generation.* The week-specific number of units donated and needed in any specific week are each generated by negative binomial stochastic processes detailed in Section ??. A *supply shock*, in this paper we consider a flu epidemic as a running example, can be introduced that reduces the number of eligible donors by a given percentage, with the reduction starting at a given calendar week and continuing for a fixed number of weeks. These percentages can be donor-group specific. At the start of the shock, the identified number of individuals enter a “flu embargo” period during which they are ineligible to donate. Subsequently, they re-enter the eligible donor population. A *demand spike* can be introduced, starting at a specified calendar week and persisting for a number of weeks, with the baseline demand rate inflated by a factor. To model increased efforts at recruiting donors to preemptively avoid a shortfall in blood supply, we introduce a *recruitment factor* by which the baseline donation rate for each donor group is inflated during a specified recruitment period.

*Accounting.* The simulator accounts for blood supply and demand and the differential between the two. Each week, each available blood unit is either used, stored, or expired. At the beginning of each week 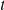, the simulator estimates the available supply for that week based on the remaining units from week 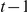, and the anticipated donation and demand in week 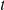. If this estimate falls outside of some given range, then the donation rate in week 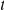 is adjusted by a factor. This is distinct from enhanced recruitment efforts described in the previous paragraph. Instead, these adjustments (which we call “regular programmatic adjustments”) are meant to reflect the real-life situation, where on a regular basis recruitment efforts may be minimally adjusted based on available supply or units may be imported from (or exported to) other collection sites in the event of shortage (or excess). A deficit occurs when the demand exceeds available supply of non-expired units. Deficits are not carried over from one week to the next.

### 3.2 Notation and Assumptions

#### 3.2.1 State Occupancy

With “
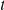
” indexing week, we use the following notation for the week-specific numbers of individuals in various states. (The total number of weeks to be simulated is specified by the user.)

R*(t) = The total number of “regular donors,” irrespective of their deferral or embargo status. Those deferred due to donation or embargoed due to “flu” cannot donate; others are eligible to donate.

S* *(t)* = The total number of “sporadic donors,” irrespective of their deferral or embargo status. Those deferred due to donation or embargoed due to “flu” cannot donate; others are eligible to donate.

N* *(t)* = The total number of “never-donors,” irrespective of their embargo status. Those embargoed due to “flu” cannot donate; others are eligible to donate.

Pop* *(t)* = 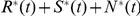, the total size of the reference population.

*R_e_ (t)* = The number of regular donors who are “eligible to donate in week 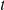
” (i.e. were not in donation deferral nor flu embargo, and did not emigrate from the system).

*S_e_ (t)* = The number of sporadic donors who are eligible to donate in week 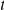.

*N_e_ (t)* = The number of never donors who are eligible to donate in week 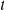.

#### 3.2.2 Stochastic Transitions

*Donations.* We assume the number of donations in week 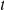 follows a negative binomial distribution (an over-dispersed Poisson) with mean and variance depending on the number of individuals eligible to donate in week 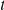. Let 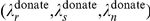 be the group-specific donation rates and 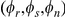 be the over-dispersion parameters. Then, the group-specific number of donations in week 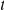 are:
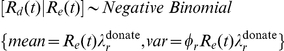


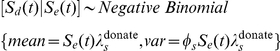


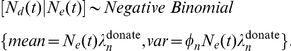
To specify an *enhanced recruitment period*, each donation rate 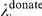 can be increased by a common factor 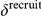 for the interval 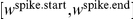. During weeks with no enhanced recruitment, regular programmatic adjustments to recruitment are made, where each donation rate 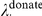 is increased (or decreased) by a common factor 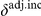 (or 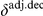) whenever estimated available supply falls outside of the range 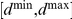, as described in Section 3.1.2.

*Demand.* Similarly, we assume the number of demanded units in week 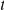 follows a negative binomial distribution with mean and variance depending on the number of individuals in the reference population [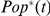] in week 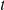. With 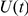 the number of units demanded in week 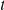, 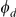 the over-dispersion parameter and 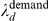 the demand rate, the number of demanded units in week 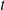 is:
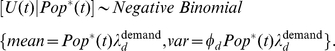
A *demand spike* is specified by inflating the baseline demand rate 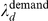 by 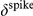 for the interval 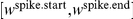.

#### 3.2.3 Deterministic State Transitions

The order of events in a given week is: add to the eligible donor pool those who have finished their deferral period (either the standard deferral period after donating, or the “flu” embargo period); embargo a fixed number due to “flu;” generate donations from the never-donor, regular and sporadic groups (see Section 3.2.2); triage first-time donors to emigrate out of the donation system, or to become regular or sporadic; defer donors for 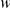 weeks; remove emigrants; add immigrants. No individual can immigrate and emigrate in the same week. Details of these transitions are in the Appendix S1; here we highlight a few.

*Embargo due to “flu”.* A flu epidemic (or similar supply shock) can operate in the interval 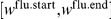. If a week is in this interval, a donor group-specific number of individuals [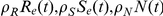] are ineligible to donate for a 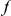-week embargo period, where the 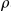 are group-specific ineligiblity proportions. A common 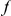 applies to all donor groups.

*First-time donor triage.* A fraction (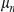) of first-time donors [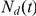] emigrate from the donation system. Of the remaining (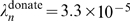) first-time donors in the system, a fixed fraction (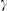) become regular donors and fraction (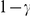) become sporadic donors.

*Immigration.* Immigration into the donation system occurs exclusively through the never-donor group. Each week, a fixed number [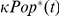] of immigrants enter the never-donor group, where 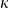 is the immigration rate.

*Emigration.* A fixed number of individuals [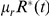, 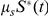, 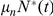] emigrate from the regular, the sporadic and the never donor groups and leave the donation system, where the 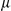 are group-specific emigration rates.

#### 3.2.4 Availability of Donated Units

There is a fixed shelf-life of 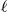 (specified by user) weeks for donated blood units.

Units can be donated and used in the same week.

Units expiring in a given week cannot be used that week.

Units are used on a “first-in, first-out” basis. For example, suppose that the shelf life 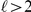, that 100 units are donated in week 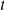; and 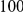 units are donated in week 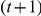; 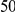 units are demanded in week 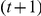 and 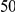 units are demanded in week 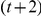. Then the supply for the 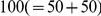 demanded units comes from the 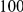 units donated in week 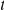, and not from those donated in week 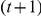.

A deficit (demand exceeds supply) is computed separately for each week; previous deficits are not carried forward. Therefore, unmet demand in week 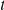 cannot be met in week 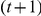. In normal circumstances, this issue could be resolved by shipping supplies from other regions.

## Results

### 4.3 Examples: Simulation Scenarios

In this section, we present sample simulator inputs and outputs to illustrate possibles uses of the simulator. We consider two principal scenarios: “normal conditions” and “moderate supply shock and demand spike,” each over a duration of 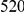 weeks. For the latter, we describe six different enhanced recruitment strategies. We note that the assumptions made in this section to determine input values are distinct from, and do not affect, the structural assumptions made for the simulator as described in Sections 3.1 and 3.2. In particular, the structural assumptions reflect certain inherent (fixed) features of the blood donation system, whereas the assumptions for our inputs are unique to a particular time-region.

Table 1 (Column 3) presents parameter values for the “normal period.” We explain our choice of values in the following text, using double square brackets [[ ]] to indicate the corresponding element in Table 1. These values are based on weekly blood donations data from 1996–2005 and on input from Red Cross staff. The blood donations data come from the American Red Cross Blood Services (ARCBS) Connecticut region (see [5]). In the ARCBS Connecticut region, the annual adult population was on average 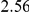 million over the ten-year period. We assume 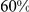 of this population is eligible to donate [6], although a recent study has shown that fewer people are actually eligible to donate [7]. Based on the 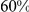 eligibility rate, we populate our simulator with 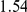 million eligible donors [[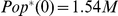]]. Our dataset includes indicators of first-time donors, which we use to estimate the first-time donation rate. In particular, we take the first-time donation rate to be the mean weekly proportion of first-time donations out of our estimated never-donor pool [[i.e. 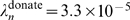 is the average over all weeks 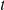 in the ten-year period, of 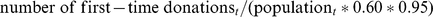]], where we assume 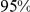 of eligible donors are never-donors, based on the estimate that 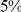 of eligible adults donate at least once a year [8]. Donation rates for the regular and sporadic groups reflect that regular donors donate 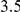 times a year [[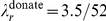]]; and that sporadic donors donate once every 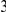 years [[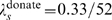]]. The deferral period and shelf-life period are specified at their standard values for red blood cell donations [[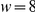 and 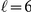, respectively]]. To initiate the simulator, we calculate steady-state values for the relative sizes of the never-donor, sporadic donor, and regular donor states [[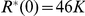, 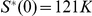, 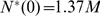]] (see the Appendix S1 for details). The advantage of setting such initial values is a shorter burn-in period before reaching a steady-state.

To estimate the mean and overdispersion parameters for the negative-binomial distributions used to generate donations, we fit a Generalized Linear Model (glm() function in R) with a log-link to the weekly donations from the Connecticut data, adjusting for age, gender, extreme events (i.e., indicators for a Red Cross staff strike, for September 11, extreme weather, and flu epidemics), and calendar time, with an offset for the age-gender-specific population. We used the model-based estimates to specify overdispersion rates for regular and sporadic donations [[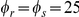]].

Of the first-time donors who do not emigrate from the system after donating, we specify the proportion 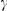 who become regular donors to be 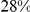 [[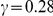]]. The remaining 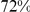 become sporadic donors. These are based on observed rates in the Connecticut data. In our data, we observed approximately equal average weekly donations and average weekly demand. The usual weekly demand rate, therefore, is set so that the average weekly demand matches approximately the average weekly donations [[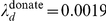]].

The immigration rate into the never-donor pool is based on the United States annual birth rate of 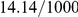 persons [9], converted to a weekly rate [[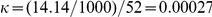]]. To maintain a closed system, in which the total population of our system is constant over time, we set the emigration rates from the never-donor, regular, and sporadic groups each equal to the immigration rate [[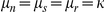]].

We specify regular programmatic adjustments to occur whenever the estimated available supply falls below 1500 units (or above 2500 units), in which case we multiply the usual donation rate in week 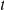 by 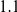 (or 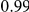) [[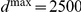, 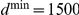, 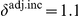, 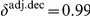]]. The range is chosen to reflect approximately two-thirds of weekly demand, and is based on unpublished data from the American Red Cross.

**Figure 2:**
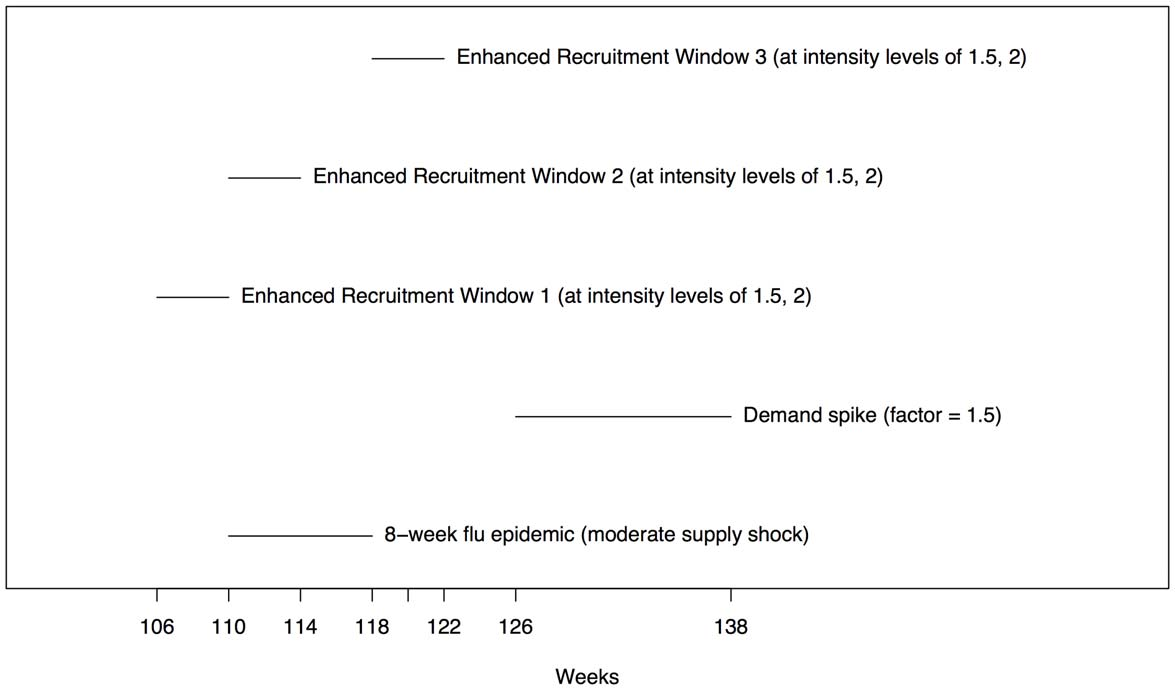
Timeline of events during a period with a moderate supply shock and a demand spike. The 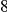-week supply shock (flu epidemic) starts in week 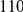 and is followed by an increase in demand by a factor of 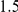 starting in week 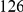, and lasting for 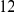 weeks. We consider three different 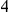-week windows of time for enhanced recruitment, each with 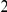 levels of intensity.

Table 2 presents additional parameter values needed to specify the hypothetical period of “moderate supply shock and demand spike.” We consider an 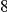-week flu period starting in week 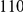 [[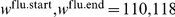]], which is followed by a spike in demand starting in week 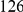, lasting for 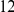 weeks [[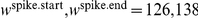]]. We specify this demand spike to reflect, for example, a spike in demand due to elective surgeries that may be postponed during a flu period. During the demand spike, we specify the usual demand rate to be increased by a multiplicative factor of 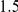 [[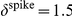]]. We assume 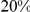 of each donor group is ineligible to donate due to flu, and are in embargo for a period of 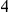 weeks [[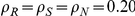, 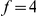]]. For enhanced recruitment strategies, we consider two intensities of increased recruitment (multiplicative factors of 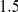 and 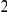 applied to the usual donation rates); and three starting times for a 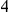-week enhanced recruitment period (starting 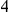 weeks prior to start of flu, week 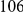; same week as start of flu, week 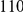; and at the end of the flu, week 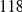), for a total of six different enhanced recruitment strategies. Figure 2 displays a timeline of key events - flu epidemic, period of demand spike, and the three different enhanced recruitment windows of time.

### 4.4 Simulation Results

**Figure 3:**
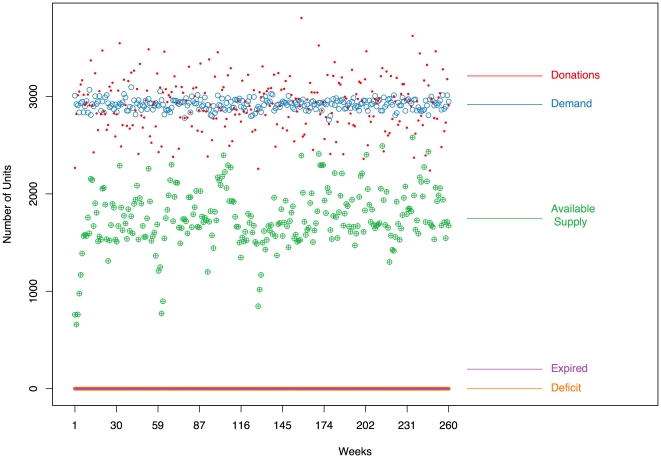
Time series of blood donations, demand, available supply, expired units, and deficit during a “Normal Period,” from one run of the simulator over 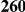 weeks after a 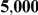-week burn-in period (not shown).

**Figure 4:**
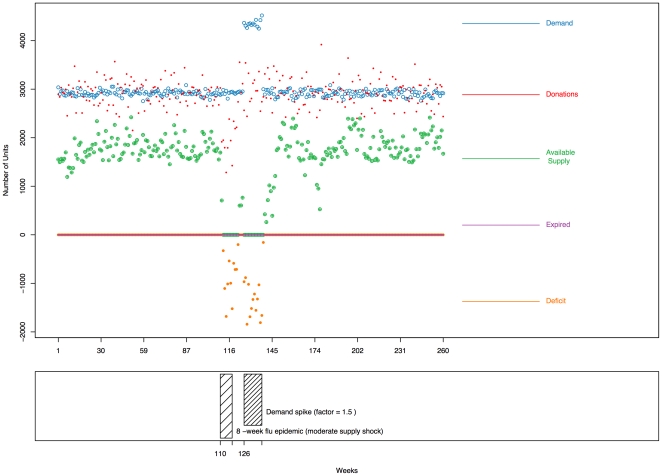
Time series of blood donations, demand, available supply, expired units, and deficit during a period with a moderate supply shock and a demand spike, with no enhanced recruitment, from one run of the simulator over 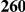 weeks after a 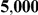-week burn-in period (not shown). The supply shock (flu epidemic) starts in Week 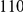 and lasts for 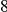 weeks, and the demand spike starts in Week 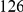 and lasts for 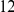 weeks, as shown in the bottom plot.

For each scenario (“normal,” “moderate supply shock” with or without recruitment strategies), we performed 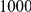 simulation runs over 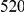 weeks, the results for which we summarize in this section. Figure 3 displays the time series (the first 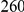 weeks of the 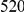 total weeks are shown), of blood patterns from one run of the simulator in a “normal” period, as described by the parameter values in Table 1. In particular, there are separate time series for each of the following weekly quantities: donations (red closed circles), demand (blue open circles), available supply (green open circles), expired units (purple open squares), and deficits (yellow closed circles). Each series is also labelled in the plots. During this normal period, we observe relatively stable patterns with some inherent variability. The weekly average number of donated units in this single run of the simulator is 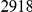, with an average available supply of 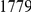 units. The average weekly demand is 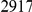 units, and on average, 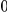 units expire each week. There are no deficit weeks, as we would expect in a normal period. Figure 4 displays the time series (the first 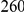 weeks of the 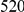 total weeks are shown) from one run under the scenario of moderate supply shock and demand spike, without any enhanced recruitment. The bottom portion of Figure 4 depicts the periods of flu (moderate supply shock) and demand spike, which we can see corresponds, in the upper plot, with decreased donations and increased demand, respectively.

To understand the effects on deficit of the recruitment strategies during the scenario with moderate supply shock and demand spike, we consider the number of weeks with a deficit and the average deficit amount in these weeks. We summarize these quantities by taking the 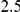 and 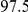 sample percentiles from the 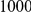 runs for each strategy (Table 3). Without enhanced recruitment, for example, the number of weeks with a deficit fell in the range of 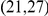 in 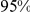 of the simulation runs; and the average deficit in these weeks with deficits fell in the range of 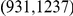 in 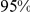 of the simulation runs. There are no significant differences in average deficit amount and number of deficit weeks across the various enhanced recruitment strategies. There does seem to be, however, a slight decrease in number of weeks with deficits if we adopt at least one of the enhanced recruitment strategies, and in particular, the strategies that recruit at intensity levels of 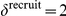.

**Table 3:** Number of Weeks with Deficit (top) and Average Deficit Amount in Weeks with Deficit (bottom) for different recruitment strategies in respose to a moderate supply shock (Weeks 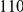
–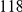) and demand spike (Weeks 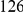
–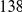).

			*(Number of weeks with Deficit)*	
			*(Average Deficit Amount in Weeks with Deficit)*	
			Multiplicative factor ( 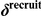 )	
			1.5	2
Weeks	([*w* ^recruit.start^, *w* ^recruit.end^])	[106, 110]	(17,24)	(12,19)
			(869, 1223)	(903,1424)
		[110, 114]	(18, 25)	(15,22)
			(897,1243)	(893, 1281)
		[118, 122]	(18,25)	(15,22)
			(915,1265)	(922,1317)
No enhancement			(21,27)	
			(931,1237)	

Six of the strategies reflect enhanced recruitment, with different starting weeks and intensities (all lasting for 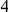 weeks); the seventh strategy labelled “No enhancement” represents no enhanced recruitment efforts. The lower and upper bounds are 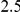 and 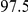-sample percentiles taken from 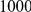 simulation runs for each strategy.

Of additional interest, we also summarize the number of weeks in which regular programmatic adjustments to recruitment were needed either because of potential excess or shortage of supply. In the scenario without any supply shocks, the (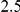, 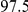)-sample quantile of number of weeks where adjustments were made to *decrease* collection was 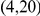. In scenarios with supply shock, regardless of enhanced recruitment strategy, the corresponding sample quantiles were similar to those from the non-supply shock scenario. The (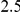, 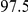)-sample quantile of number of weeks where adjustments were made to *increase* collection was 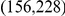 during the scenario without a supply shock. In scenarios with supply shock and any enhanced recruitment, the corresponding sample quantiles ranged from 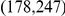 to 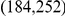; whereas in the absence of enhanced recruitment, the corresponding quantile was slightly higher at 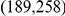.

### 4.5 Extreme Strategies

The reported scenarios highlight the fine-tuning needed to reduce the likelihood or extent of a supply deficit. Our results suggest that *timing* of enhanced recruitment may be key to attenuating effects. To investigate this idea further, we explored “extreme” enhanced recruitment strategies, for example, extended durations of recruitment period and higher intensities of enhanced recruitment. We found that, under the assumptions of our simulation system, specifying an enhanced recruitment period slightly before the start of, and lasting throughout the duration of, a demand spike seemed to more significantly reduce deficit effects relative to the six plausible strategies we considered. Specifically, for example, for an enhanced recruitment period starting at the end of the flu period (week 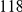) yet before the demand spike, and lasting through the duration of the demand spike to week 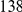, with recruitment increasing by a factor of 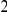 (which we refer to as Extreme Strategy 1), the (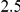, 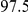)-sample quantiles of number of weeks with deficit and average deficit amount in those weeks were 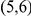 and 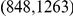, respectively, representing a drop in average number of weeks with deficit relative to a shorter duration of enhanced recruitment. As a more extreme case with respect to timing, we considered an enhanced recruitment start week before the start of flu and lasting through the duration of the demand spike (i.e. weeks 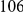
–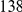), at an intensity of 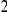 (Extreme Strategy 2). This resulted in no deficit at all in any of the 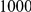 simulation runs. In contrast, an enhanced recruitment strategy during weeks 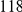
–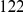, the same 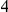-week window as two of the six strategies we considered in Section 4.3, but with recruitment increasing by an extreme factor of 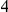 (Extreme Strategy 3), the (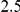, 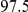)-sample quantiles of number of weeks with deficit and average deficit amount in those weeks were 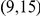 and 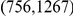, respectively. This latter strategy (Extreme Strategy 3) characterized by extreme *intensity* does not yield as marked an impact on deficit characteristics as the previous two (Extreme Strategies 1 and 2), which are characterized by extreme *timing*. We label these all as “extreme” strategies since of course in reality, the system is generally subject also to resource and staff constraints. Although, we note that such strategies could be employed over relatively remotely located, different geographic areas. We also highlight that the donation rates are applied to the number of eligible donors not in embargo (due to flu or due to donation). In weeks with flu embargoes, this pool of eligible donors not in embargo is smaller than during normal periods. So a recruitment factor of 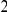 (or 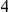), for example, may not directly translate into a doubling (or quadrupling) of the average weekly donations during the recruitment period relative to non-recruitment periods. As such, some of these “extreme” scenarios may not be entirely unrealistic. Regardless, they suggest the importance of timing, and demonstrate that effective timing depends on anticipating shocks (a difficult task) or rapidly implementing recruitment efforts (a possible task if systems are in place). Fortunately, under the assumptions of our simulation system, the ability to attenuate deficits reflects a degree of robustness and elasticity in the blood donation system. Since the majority of eligible adults never have donated, during times of crises, even a small increase in the donation rate among this large pool of eligibles, subject to resource and staff constraints, can attenuate a shortfall.

## Discussion

We have developed a basic simulation system designed to study the normal weekly variation in donated, required and available blood units. Though it does not incorporate all features of an actual donation system, it does allow control of a wide variety of parameters and the ability to instigate a supply shock, a demand spike, and enhanced recruiting. We have illustrated features of the simulator by running several scenarios with these features, but it can be used for other planning purposes or simply to understand the blood donation flow, under a variety of “normal” parameter specifications. Of note, the basic simulator is not designed to manage blood bank inventories, but rather is developed to quantitatively describe a blood supply system so that changes to various parameters of such a system can be evaluated for their impact on the system.

We have considered two principal scenarios and potential responses to the scenario with moderate supply shock and demand spike. By specifying different input values for the simulator, one can examine other scenarios and responses. Another feature, shared by many stochastic systems, of our simulator is that it identifies the sensitivies of the results to input values, pointing to the importance of good knowledge of inputs.

We use 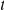 to index weeks, but no structural changes are needed to move to day-specific accounting. The parameter values we use are relevant for red blood cell donations, however all values can be modified to reflect donations of other blood products. These require changes in donation rates, the deferral periods and shelf life. The current parameter values do not reflect a demand decrease due to flu, but could be easily modified to account for this. With additional programming, structural enhancements can be implemented. The time-homogeneous, negative binomial donation rates can be replaced by season-specific rates. If the time scale is days, the delay between donation and use of platelets (or red blood cells) can be incorporated. To accommodate age-specific or gender-specific donation rates, the simulator can be run for several age/gender strata and the output combined. Enhancements could allow for age and gender specific rates and “aging” within a single simulation system. The current simulator allows for one time interval for a supply shock and one for a demand spike. Enhancements could allow for multiple intervals or for a Markov transition model between the normal and the extreme rates, producing “episodes” of extreme conditions. Another enhancement could allow for different demand rates to be specified during periods of supply shock. This would allow for examination of the impact of policies aimed at decreasing use in response to a supply shock. Still another enhancement could allow for a shorter donation-related deferral period during times of need. Finally, as indicated in the Introduction, the basic model presented in this paper simulates the overall supply and demand of red blood cells or whole blood. The supply and demand scenarios for red blood cells can vary significantly among different blood groups, for example, blood type “O” versus type “A.” There could be a deficit for type “O” red blood cells while units of type “A” could be expiring, because the blood type composition of donations (supply) does not match with that of transfusion demand and also type “O” red blood cells can be used for recipients of different blood types in emergency situations. Further, rare blood groups are present in much smaller numbers such that the supply and demand could be subject to much greater proportional swings. The simulator could be enhanced to allow for type-specific donation and demand rates.

A previous blood supply model was developed by Custer et al. [10]. A key feature of the Custer model is its ability to help assess the impact of predonation deferrals and demographic differences between donors on blood supply, and evaluate blood safety and policy decisions. It is built around data collected by Blood Centers of the Pacific, and incorporates cost estimates. A key feature of our model is its ability to model the potential effects of extreme events during some user-specified timing, and evaluate various response recruitment strategies. Several features which distinguish it from the Custer model include week (or any other unit of time)-specific accounting of blood units, breakdown of donor groups into regular and sporadic, immigration and emigration from the donor system, and embargo due to unusual events during specified time periods. Our code is also available for use or modifications.

In summary, we have developed a basic simulator and illustrated example inputs and outputs. In the process we have identified strategies to consider that may increase availability of donated blood units. The simulator can be enhanced, but in its current form does serve as a useful tool for understanding the impact on the blood supply of extreme and unusual events such as a flu epidemic and how we may better prepare for and react to these threats in order to ensure blood availability.

## Supporting Information

Appendix S1 (PDF)
