# Supplementary material for: A Stochastic Simulator of a Blood Product Donation Environment with Demand Spikes and Supply Shocks
Source: PLoS One. 2011 Jul 26;6(7):e21752. doi: 10.1371/journal.pone.0021752 (PMC3144207; doi:10.1371/journal.pone.0021752)
Supplement: Appendix S1 — (PDF) [file pone.0021752.s001.pdf]

# APPENDIX S1 for:

# A stochastic simulator of a blood product donation environment with demand spikes and supply shocks

Ming-Wen An, Ph.D.[1,2,†,*], Nicholas G. Reich, Ph.D.[2,3,†], Stephen O. Crawford, Ph.D.[3,4,†], Ron Brookmeyer, Ph.D.[5], Thomas A. Louis, Ph.D.[2], Kenrad E. Nelson, M.D.[3]

## 1 Appendices

### 1.1 Calculating Initial Values for a Steady State

In this section, we describe choosing initial values of the respective donor group populations based on a steady-state. The steady-state is characterized by no change in donor-group population sizes over time. The advantage of choosing initial values in this way is a shorter burn-in period, which is the number of iterations of the simulator that are needed before reaching a steady-state. We assume a closed system, meaning the immigration and emigration rates are equal so that the total population of the entire system is constant over time. The rates of change in respective donor group population sizes are described by the following relations:

$$\begin{aligned} N'(t) &= Pop^*(t)\kappa - N^*(t)[\lambda_n^{\text{donate}} + \mu_n] \\ R'(t) &= \lambda_n^{\text{donate}}(1-\mu_n)\gamma N^*(t) - R(t)\mu_r \\ S'(t) &= \lambda_n^{\text{donate}}(1-\mu_n)(1-\gamma) N^*(t) - S(t)\mu_s \end{aligned}$$

A steady-state in which the respective donor group population sizes remain constant over time can be obtained by setting the above equations to 0 (equivalent to there being no change in population sizes over time). We take the total population size $Pop^*(t)$, $\kappa = \mu_n = \mu_r = \mu_s$, and $\lambda_n^{\text{donate}}$ as fixed, and solve for values of $N^*(t)$, $R^*(t)$, and $S^*(t)$, which we take to be our initial donor group population sizes.

$$\begin{aligned} N'(t) &= Pop^*(t)\kappa - N^*(t)[\lambda_n^{\text{donate}} + \mu_n] = 0 \\ R'(t) &= \lambda_n^{\text{donate}}(1-\mu_n)\gamma N^*(t) - R(t)\mu_r = 0 \\ S'(t) &= \lambda_n^{\text{donate}}(1-\mu_n)(1-\gamma) N^*(t) - S(t)\mu_s = 0 \end{aligned}$$

The resulting solutions are:

$$
\begin{aligned}
N^*(t) &= Pop^*(t)\left(\frac{\kappa}{\lambda_n^{\text{donate}} + \mu_n}\right) \\
R^*(t) &= Pop^*(t)\left(\frac{\kappa}{\lambda_n^{\text{donate}} + \mu_n}\right)\left(\frac{\lambda_n^{\text{donate}}(1-\mu_n)\gamma}{\mu_r}\right) \\
S^*(t) &= Pop^*(t)\left(\frac{\kappa}{\lambda_n^{\text{donate}} + \mu_n}\right)\left(\frac{\lambda_n^{\text{donate}}(1-\mu_n)(1-\gamma)}{\mu_s}\right).
\end{aligned}
$$

## 1.2 Detailed Notation

The following lists all of the week-specific tallies that are needed for the simulator, and to reflect its detailed structure. However, these are not needed to understand the overall flow of units.

$R^*(t) =$ The total number of "regular donors," irrespective of their deferral or embargo status. Those deferred due to donation or embargoed due to "flu" cannot donate; others are eligible to donate.

$S^*(t) =$ The total number of "sporadic donors," irrespective of their deferral or embargo status. Those deferred due to donation or embargoed due to "flu" cannot donate; others are eligible to donate.

$N^*(t) =$ The total number of "never-donors," irrespective of their deferral or embargo status. Those deferred due to donation or embargoed due to "flu" cannot donate; others are eligible to donate.

$Pop^*(t) =$ $R^*(t) + S^*(t) + N^*(t)$, the total size of the reference population in week $t$.

$R_e(t) =$ The number of regular donors who are eligible to donate in week $t$ (were not in donation deferral nor flu embargo, and did not emigrate from the system).

$S_e(t) =$ The number of sporadic donors who are eligible to donate in week $t$ (were not in donation deferral nor flu embargo, and did not emigrate from the system).

$N_e(t) =$ The number of never donors who were eligible to donate in week $t$ (were not in flu embargo, and did not emigrate from the system).

$Re(t) =$ The number of regular donors who were in the deferral period in week $(t-1)$, but who are eligible to donate in week $t$. They are included in $R_e(t)$, but have a specific term for accounting purposes.

$Se(t) =$ The number of sporadic donors who were in the deferral period in week $(t-1)$, but who are eligible to donate in week $t$. They are included in $S_e(t)$, but have a specific term for accounting purposes.

$X_R^{out}(t) =$ The number of regular donors who are not eligible to donate due to flu or other syndromes in week $t$.

$X_S^{out}(t) =$ The number of sporadic donors who are not eligible to donate due to flu or other syndromes in week $t$.

$X_N^{out}(t) =$ The number of never-donors who are not eligible to donate due to flu or other syndromes in week $t$.

$X_R^{in}(t) =$ The number of regular donors who are again eligible to donate in week $t$, but who were ineligible in week $(t-1)$ due to flu or other syndromes.

$X_S^{in}(t) =$ The number of sporadic donors who are again eligible to donate in week $t$, but who were ineligible in week $(t-1)$ due to flu or other syndromes.

$X_N^{in}(t) =$ The number of never-donors who are again eligible to donate in week $t$, but who were ineligible in week $(t-1)$ due to flu or other syndromes.

$R_d(t) =$ The number of regular donors who donate a unit of blood in week $t$.

$S_d(t) =$ The number of sporadic donors who donate a unit of blood in week $t$.

$N_d(t) =$ The number of first-time donors who donate a unit of blood in week $t$.

During week $t$, the week-specific numbers of "units" of blood products are:

$Y_s(t) =$ The number of units available in week $t$ from the batch donated in week $s$. See Section 1.4 for detailed accounting.

$U(t) =$ The number of units demanded in week $t$.

$D(t) =$ The number of units expired in week $t$.

$A(t) =$ The number of units available by the end of week $t$ (after units have expired, been used, or been newly-donated in week $t$).

To specify recruitment efforts as part of regular programmatic planning efforts, we specify the following:

$d^{\text{max}} =$ The maximum threshold of available supply; if projected available supply falls above this threshold, then adjustments to recruitment are made to decrease donations.

$d^{\text{min}} =$ The minimum threshold of available supply; if projected available supply falls below this threshold, then adjustments to recruitment are made to increase donations.

$\delta^{\text{adj.dec}} =$ The factor ($< 1$) by which usual donation rates (common across the three donor groups) are multiplied in weeks where projected available supply falls above $d^{max}$.

$\delta^{\text{adj.inc}} =$ The factor ($> 1$) by which usual donation rates (common across the three donor groups) are multiplied in weeks where projected available supply falls below $d^{min}$.

To specify recruitment efforts, supply shocks, or demand spikes, we specify the following:

$\delta^{\text{recruit}} =$ "Recruitment" factor, factor by which the usual donation rates (common across the three donor groups) are multiplied during a user-specified recruitment period.

$w^{\text{recruit.start}} =$ The starting week of recruitment period.

$w^{\text{recruit.end}} =$ The ending week of recruitment period.

$\delta^{\text{spike}} =$ Multiplication factor to be applied to the usual demand rates (common across the three donor groups) during a user-specified period of demand spike (or drop).

$w^{\text{spike.start}} =$ The starting week of increased demand (demand spike).

$w^{\text{spike.end}} =$ The ending week of increased demand (demand spike).

$\rho_R =$ The proportion of regular donors ineligible to donate due to flu for a "flu embargo" period of $f$ weeks.

$\rho_S =$ The proportion of sporadic donors ineligible to donate due to flu for a "flu embargo" period of $f$ weeks.

$\rho_N =$ The proportion of never-donors ineligible to donate due to flu for a "flu embargo" period of $f$ weeks.

$f =$ The number of weeks of the "flu embargo" period.

## 1.3 Donation Accounting

In this section, we describe the accounting relationships for donation, immigration, and emigration. Additional details on accounting are available as part of the web supplement. We assume that no one is in the deferral nor the embargo state at $t = 0$, i.e. $R^*(0) = R_e(0), S^*(0) = S_e(0), N^*(0) = N_e(0)$.

$$\begin{aligned}
R_e(t+1) &= R_e(t) - R_d(t) + Re(t+1) - \mu_r[R^*(t)] + X_R^{in}(t+1) - X_R^{out}(t) \\
S_e(t+1) &= S_e(t) - S_d(t) + Se(t+1) - \mu_s[S^*(t)] + X_S^{in}(t+1) - X_S^{out}(t) \\
N_e(t+1) &= N_e(t) - N_d(t) + \kappa[Pop^*(t)] - \mu_n[N^*(t)] + X_N^{in}(t+1) - X_N^{out}(t) \\
Re(t+1) &= R_d(t-w) + \gamma(1-\mu_n)N_d(t-w) \\
Se(t+1) &= S_d(t-w) + (1-\gamma)(1-\mu_n)N_d(t-w) \\
X_R^{in}(t+1) &= X_R^{out}(t-f) \\
X_S^{in}(t+1) &= X_S^{out}(t-f) \\
X_N^{in}(t+1) &= X_N^{out}(t-f) \\
X_R^{out}(t+1) &= \rho_R R_e(t+1) \\
X_S^{out}(t+1) &= \rho_S S_e(t+1) \\
X_N^{out}(t+1) &= \rho_N N_e(t+1) \\
R^*(t+1) &= R^*(t) + \gamma(1-\mu_n)N_d(t) - \mu_r[R^*(t)] \\
S^*(t+1) &= S^*(t) + (1-\gamma)(1-\mu_n)N_d(t) - \mu_s[S^*(t)] \\
N^*(t+1) &= N^*(t) - N_d(t) + \kappa[Pop^*(t)] - \mu_n[N^*(t) - N_d(t)] \\
Pop^*(t+1) &= R^*(t+1) + S^*(t+1) + N^*(t+1)
\end{aligned}$$

## 1.4 Supply and Demand Accounting

- For $s = t$

  1. If $Y_{s-1}(t) > 0$, then $Y_s(t) = R_d(t) + S_d(t) + N_d(t)$
  2. If $Y_{s-1}(t) < 0$, then $Y_s(t) = R_d(t) + S_d(t) + N_d(t) + Y_{s-1}(t)$
  3. If $Y_{s-1}(t) = 0$ and $Y_{s-1}(t-1) \leq 0$, then $Y_s(t) = R_d(t) + S_d(t) + N_d(t) - U(t)$
  4. If $Y_{s-1}(t) = 0$ and $Y_{s-1}(t-1) > 0$, then $Y_s(t) = R_d(t) + S_d(t) + N_d(t)$

- For $s < t < s + \ell - 1$

  - If $Y_s(t-1) \leq 0$ then $Y_s(t) = 0$.
  - Else

    1. If $Y_{s-1}(t) > 0$, then $Y_s(t) = Y_s(t-1)$
    2. If $Y_{s-1}(t) < 0$, then $Y_s(t) = Y_s(t-1) + Y_{s-1}(t)$

       **Note**: If $Y_{s-1}(t) < 0$, then $-Y_{s-1}(t)$ represents the demand in week $t$ not covered by the batch donated in the week prior to $s$
    3. If $Y_{s-1}(t) = 0$ and $Y_{s-1}(t-1) \leq 0$, then $Y_s(t) = Y_s(t-1) - U(t)$
    4. If $Y_{s-1}(t) = 0$ and $Y_{s-1}(t-1) > 0$, then $Y_s(t) = Y_s(t-1)$

       **Note**: We distinguish between when the previously donated batch exactly met this week's demand (3), and when the previously donated batch had run out prior to this week (4)

- For $t = s + \ell - 1$ (Note: In week $s + \ell - 1$, the first batch from which demand is supplied comes from the batch donated in week $s$.)

  - If $Y_s(t-1) > 0$, then $Y_s(t) = Y_s(t-1) - U(t)$
  - If $Y_s(t-1) \leq 0$, then $Y_s(t) = 0$

- For $t < s$ and $t \geq s + \ell$, $Y_s(t) = 0$

We have the following additional accounting:

$$D(t) = \max\{0, Y_{t-\ell}(t-1)\}$$

$$A(t) = \max\{0, A(t-1) + R_d(t) + S_d(t) + N_d(t) - D(t) - U(t)\}$$

**Note:** A deficit occurs in week $t$ if $Y_t(t) < 0$ since the batch donated in week $t$ is the last "eligible" batch to be used in week $t$. Equivalently, $U(t) > A(t-1) + R_d(t) + S_d(t) + N_d(t) - D(t)$.

## 1.5 Accounting Verification

In this section, we verify the accounting definitions from Section 1.3 by "removing recursions," that is, by re-expressing quantities in non-recursive terms. We show this for the simple case of $w = 2$ and $f = 1$. Generalizations are straightforward.

<u>All Donors.</u>

Note we assume $R^*(0) = R_e(0)$ and $S^*(0) = S_e(0)$, which implies $R_d(t) = S_d(t) = 0$ for $-w \leq t < 0$. Recall $N^*(t) = N_e(t)\ \forall\ t$.

For $t = 0$, $Pop^*(0) = R^*(0) + S^*(0) + N^*(0) = R_e(0) + S_e(0) + N_e(0) = Pop(0)$.

For $t = 1$,

$$\begin{aligned}
& R^*(1) + S^*(1) + N^*(1) \\
= \;& \{R_e(0) + \gamma(1-\mu_n)N_d(0) - \mu_r[R_e(0)]\} \\
& +\{S_e(0) + (1-\gamma)(1-\mu_n)N_d(0) - \mu_s[S_e(0)]\} \\
& +\{N_e(0) - N_d(0) + \kappa[R_e(0) + S_e(0) + N_e(0)] - \mu_n[N_e(0) - N_d(0)]]\} \\
= \;& R_e(0) + S_e(0) + N_e(0) + \kappa[R_e(0) + S_e(0) + N_e(0)] \\
& -\mu_r[R_e(0)] - \mu_s[S_e(0)] - \mu_n[N_e(0)]
\end{aligned}$$

For general $t$,

$$\begin{aligned}
& R^*(t+1) + S^*(t+1) + N^*(t+1) \\
= \;& R^*(t) + \gamma(1-\mu_n)N_d(t) - \mu_r[R^*(t)] \\
& +S^*(t) + (1-\gamma)(1-\mu_n)N_d(t) - \mu_s[S^*(t)] \\
& +N^*(t) - N_d(t) + \kappa[R^*(t) + S^*(t) + N^*(t)] - \mu_n[N^*(t) - N_d(t)] \\
= \;& R^*(t) + S^*(t) + N^*(t) + \kappa[R^*(t) + S^*(t) + N^*(t)] \\
& -\mu_r[R^*(t)] - \mu_s[S^*(t)] - \mu_n[N^*(t)]
\end{aligned}$$

<u>Never-Donors.</u>

For $t = 0,\ N_e(0) = N^*(0)$.

For $t = 1$,

$$\begin{aligned} N_e(1) &= N_e(0) - N_d(0) + \kappa Pop^*(0) - \mu_n[N^*(0)] + X_N^{in}(1) - X_N^{out}(0) \\ N^*(1) &= N^*(0) - (1-\mu_n)N_d(0) + \kappa Pop^*(0) - \mu_n[N^*(0) - N_d(0)] - \mu_n N_d(0) \end{aligned}$$

For $t = 2$,

$$\begin{aligned} N_e(2) &= \{N_e(1)\} - N_d(1) + \kappa Pop^*(1) - \mu_n[N^*(1)] + X_N^{in}(2) - X_N^{out}(1) \\ &= \{N_e(0) - N_d(0) + \kappa Pop^*(0) - \mu_n[N^*(0)] + X_N^{in}(1) - X_N^{out}(0)\} \\ &\quad -N_d(1) + \kappa Pop^*(1) - \mu_n[N^*(1)] + X_N^{in}(2) - X_N^{out}(1) \\ &= N^*(0) - N_d(0) - N_d(1) + \kappa Pop^*(0) + \kappa Pop^*(1) \\ &\quad +X_N^{in}(1) - X_N^{out}(0) + \underline{X_N^{in}(2)} - X_N^{out}(1) \\ &\quad -\mu_n[N^*(0)] - \mu_n[N^*(1)] \\ &= N^*(0) - N_d(0) - N_d(1) + \kappa Pop^*(0) + \kappa Pop^*(1) \\ &\quad +X_N^{out}(-1) - X_N^{out}(0) + \underline{X_N^{out}(0)} - X_N^{out}(1) \\ &\quad -\mu_n[N^*(0)] - \mu_n[N^*(1)] \\ &= N^*(0) - N_d(0) - N_d(1) - X_N^{out}(1) \\ &\quad +\kappa Pop^*(0) + \kappa Pop^*(1) \\ &\quad -\mu_n[N^*(0)] - \mu_n[N^*(1)] \\ N^*(2) &= \{N^*(1)\} - (1-\mu_n)N_d(1) + \kappa Pop^*(1) - \mu_n[N^*(1) - N_d(1)] - \mu_n N_d(1) \\ &= \{N^*(0) - (1-\mu_n)N_d(0) + \kappa Pop^*(0) - \mu_n[N_e(0) - N_d(0)] - \mu_n N_d(0)\} \\ &\quad -(1-\mu_n)N_d(1) + \kappa Pop^*(1) - \mu_n[Nd^*(1) - N_d(1)] - \mu_n N_d(1) \\ &= N^*(0) - N_d(0) - N_d(1) + \kappa Pop^*(0) + \kappa Pop^*(1) \\ &\quad -\mu_n[N^*(0) - N_d(0)] - \mu_n[N^*(1) - N_d(1)] \end{aligned}$$

For $t = 3$,

$$\begin{aligned}
N_e(3) &= \{N_e(2)\} - N_d(2) + \kappa Pop^*(2) - \mu_n[N^*(2)] + X_N^{in}(3) - X_N^{out}(2) \\
&= \{N^*(0) - N_d(0) - N_d(1) - X_N^{out}(1) + \kappa Pop^*(0) + \kappa Pop^*(1) \\
&\quad -\mu_n[N^*(0)] - \mu_n[N^*(1)]\} \\
&\quad -N_d(2) + \kappa Pop^*(2) - \mu_n[N^*(2)] + \underline{X_N^{in}(3)} - X_N^{out}(2) \\
&= \{N^*(0) - N_d(0) - N_d(1) - X_N^{out}(1) + \kappa Pop^*(0) + \kappa Pop^*(1) \\
&\quad -\mu_n[N^*(0)] - \mu_n[N^*(1)]\} \\
&\quad -N_d(2) + \kappa Pop^*(2) - \mu_n[N^*(2)] + \underline{X_N^{out}(1)} - X_N^{out}(2) \\
&= N^*(0) - N_d(0) - N_d(1) - N_d(2) - X_N^{out}(2) \\
&\quad +\kappa Pop^*(0) + \kappa Pop^*(1) + \kappa Pop^*(2) \\
&\quad -\mu_n[N^*(0)] - \mu_n[N^*(1)] - \mu_n[N^*(2)] \\
N^*(3) &= \{N^*(2)\} - (1-\mu_n)N_d(2) + \kappa Pop^*(2) - \mu_n[N^*(2) - N_d(2)] - \mu_n N_d(2) \\
&= \{N^*(0) - N_d(0) - N_d(1) + \kappa Pop^*(0) + \kappa Pop^*(1) \\
&\quad -\mu_n[N^*(0) - N_d(0)] - \mu_n[N^*(1) - N_d(1)]\} \\
&\quad -N_d(2) + \kappa Pop^*(2) - \mu_n[N^*(2) - N_d(2)] \\
&= N^*(0) - N_d(0) - N_d(1) - N_d(2) \\
&\quad +\kappa Pop^*(0) + \kappa Pop^*(1) + \kappa Pop^*(2) \\
&\quad -\mu_n[N^*(0) - N_d(0)] - \mu_n[N^*(1) - N_d(1)] - \mu_n[N^*(2) - N_d(2)]
\end{aligned}$$

For $t = 4$,

$$\begin{aligned}
N_e(4) &= \{N_e(3)\} - N_d(3) + \kappa Pop^*(3) - \mu_n[N^*(3)] + X_N^{in}(4) - X_N^{out}(3) \\
&= \{N^*(0) - N_d(0) - N_d(1) - N_d(2) - X_N^{out}(2) \\
&\quad +\kappa Pop^*(0) + \kappa Pop^*(1) + \kappa Pop^*(2) \\
&\quad -\mu_n[N^*(0)] - \mu_n[N^*(1)] - \mu_n[N^*(2)]\} \\
&\quad -N_d(3) + \kappa Pop^*(3) - \mu_n[N^*(3)] + \underline{X_N^{in}(4)} - X_N^{out}(3) \\
&= \{N^*(0) - N_d(0) - N_d(1) - N_d(2) - X_N^{out}(2) \\
&\quad +\kappa Pop^*(0) + \kappa Pop^*(1) + \kappa Pop^*(2) \\
&\quad -\mu_n[N^*(0)] - \mu_n[N^*(1)] - \mu_n[N^*(2)]\} \\
&\quad -N_d(3) + \kappa Pop^*(3) - \mu_n[N^*(3)] + \underline{X_N^{out}(2))} - X_N^{out}(3) \\
&= N^*(0) - N_d(0) - N_d(1) - N_d(2) - N_d(3) - X_N^{out}(3) \\
&\quad +\kappa Pop^*(0) + \kappa Pop^*(1) + \kappa Pop^*(2) + \kappa Pop^*(3) \\
&\quad -\mu_n[N^*(0)] - \mu_n[N^*(1)] - \mu_n[N^*(2)] - \mu_n[N^*(3)] \\
N^*(4) &= \{N^*(3)\} - (1-\mu_n)N_d(3) + \kappa Pop^*(3) - \mu_n[N^*(3) - N_d(3)] - \mu_n N_d(3) \\
&= \{N^*(0) - N_d(0) - N_d(1) - N_d(2) \\
&\quad +\kappa Pop^*(0) + \kappa Pop^*(1) + \kappa Pop^*(2) \\
&\quad -\mu_n[N^*(0) - N_d(0)] - \mu_n[N^*(1) - N_d(1)] - \mu_n[N^*(2) - N_d(2)]\} \\
&\quad -N_d(3) + \kappa Pop^*(3) - \mu_n[N^*(3) - N_d(3)] \\
&= \{N^*(0) - N_d(0) - N_d(1) - N_d(2) - N_d(3) \\
&\quad +\kappa Pop^*(0) + \kappa Pop^*(1) + \kappa Pop^*(2) + \kappa Pop^*(3) \\
&\quad -\mu_n[N^*(0) - N_d(0)] - \mu_n[N^*(1) - N_d(1)] - \mu_n[N^*(2) - N_d(2)] - \mu_n[N^*(3) - N_d(3)]\}
\end{aligned}$$

<u>Regular Donors. (Analogous for Sporadic Donors)</u>

For $t = 0$, $R_e(0) = R^*(0)$.

For $t = 1$,

$$\begin{aligned} R_e(1) &= R_e(0) - R_d(0) + Re(1) - \mu_r[R^*(0)] + X_R^{in}(1) - X_R^{out}(0) \\ R^*(1) &= R^*(0) + \gamma N_d(0) - \mu_r[R^*(0)] \end{aligned}$$

For $t = 2$,

$$\begin{aligned} R_e(2) &= \{R_e(1)\} - R_d(1) + Re(2) - \mu_r[R^*(1) + \gamma N_d(1)] + X_R^{in}(2) - X_R^{out}(1) \\ &= \{R_e(0) - R_d(0) + \underline{Re(1)} - \mu_r[R^*(0)x] + X_R^{in}(1) - X_R^{out}(0)\} \\ &\quad -R_d(1) + \underline{Re(2)} - \mu_r[R^*(1)] + X_R^{in}(2) - X_R^{out}(1) \\ &= R_e(0) - R_d(0) + \underline{R_d(-2) + \gamma(1-\mu_n)N_d(-2)} - \mu_r[R^*(0)]\} \\ &\quad -R_d(1) + \underline{R_d(-1) + \gamma(1-\mu_n)N_d(-1)} - \mu_r[R^*(1)] \\ &\quad +X_R^{in}(1) - X_R^{out}(0) + X_R^{in}(2) - X_R^{out}(1) \\ &= R_e(0) - R_d(0) + R_d(-2) + \gamma(1-\mu_n)N_d(-2) - \mu_r[R^*(0)]\} \\ &\quad -R_d(1) + R_d(-1) + \gamma(1-\mu_n)N_d(-1) - \mu_r[R^*(1)] \\ &\quad +\underline{X_R^{in}(1)} - X_R^{out}(0) + \underline{X_R^{in}(2)} - X_R^{out}(1) \\ &= R_e(0) - R_d(0) + R_d(-2) + \gamma(1-\mu_n)N_d(-2) - \mu_r[R^*(0)]\} \\ &\quad -R_d(1) + R_d(-1) + \gamma(1-\mu_n)N_d(-1) - \mu_r[R^*(1)] \\ &\quad +\underline{X_R^{out}(-1)} - X_R^{out}(0) + \underline{X_R^{out}(0)} - X_R^{out}(1) \\ &= R_e(0) - R_d(0) - R_d(1) - \mu_r[R^*(0)] - \mu_r[R^*(1)] - X_R^{out}(1) \\ R^*(2) &= \{R^*(1)\} + \gamma(1-\mu_n)N_d(1) - \mu_r[R^*(1)] \\ &= \{R^*(0) + \gamma(1-\mu_n)N_d(0) - \mu_r[R^*(0)]\} \\ &\quad +\gamma(1-\mu_n)N_d(1) - \mu_r[R^*(1)] \\ &= R_e(0) + \gamma(1-\mu_n)[N_d(0) + N_d(1)] \\ &\quad -\mu_r[R^*(0)] - \mu_r[R^*(1)] \end{aligned}$$

For $t = 3$,

$$\begin{aligned}
R_e(3) &= \{R_e(2)\} - R_d(2) + Re(3) - \mu_r[R^*(2)] + X_R^{in}(3) - X_R^{out}(2) \\
&= \{R_e(0) - R_d(0) - R_d(1) - \mu_r[R^*(0)] - X_R^{out}(1)\} \\
&\quad -R_d(2) + \underline{Re(3)} - \mu_r[R^*(2)] + X_R^{in}(3) - X_R^{out}(2) \\
&= \{R_e(0) - R_d(0) - R_d(1) - \mu_r[R^*(0)] \\
&\quad -\mu_r[R^*(1)] - X_R^{out}(1)\} \\
&\quad -R_d(2) + \underline{R_d(0) + \gamma(1-\mu_n)N_d(0)} - \mu_r[R^*(2)] + X_R^{in}(3) - X_R^{out}(2) \\
&= \{R_e(0) - R_d(0) - R_d(1) - \mu_r[R^*(0)] \\
&\quad -\mu_r[R^*(1)] - X_R^{out}(1)\} \\
&\quad -R_d(2) + R_d(0) + \gamma(1-\mu_n)N_d(0) - \mu_r[R^*(2)] + \underline{X_R^{in}(3)} - X_R^{out}(2) \\
&= \{R_e(0) - R_d(0) - R_d(1) - \mu_r[R^*(0)] \\
&\quad -\mu_r[R^*(1)] - X_R^{out}(1)\} \\
&\quad -R_d(2) + R_d(0) + \gamma(1-\mu_n)N_d(0) - \mu_r[R^*(2)] + \underline{X_R^{out}(1)} - X_R^{out}(2) \\
&= \{R_e(0) - \mu_r[R^*(0)] \\
&\quad -R_d(1) - \mu_r[R^*(1)]\} \\
&\quad -R_d(2) + \gamma(1-\mu_n)N_d(0) - \mu_r[R^*(2)] - X_R^{out}(2) \\
&= R_e(0) - R_d(1) - R_d(2) + \gamma N_d(0) \\
&\quad -\mu_r[R^*(0)] - \mu_r[R^*(1)] - \mu_r[R^*(2)] - X_R^{out}(2) \\
R^*(3) &= \{R^*(2)\} + \gamma(1-\mu_n)N_d(2) - \mu_r[R^*(2)] \\
&= \{R^*(0) + \gamma(1-\mu_n)N_d(0) - \mu_r[R^*(0)] \\
&\quad +\gamma(1-\mu_n)N_d(1) - \mu_r[R^*(1)]\} \\
&\quad +\gamma(1-\mu_n)N_d(2) - \mu_r[R^*(2)] \\
&= R_e(0) + \gamma(1-\mu_n)[N_d(0) + N_d(1) + N_d(2)] \\
&\quad -\mu_r[R^*(0)] - \mu_r[R^*(1)] - \mu_r[R^*(2)]
\end{aligned}$$

For $t = 4$,

$$\begin{aligned}
R_e(4) &= \{R_e(3)\} - R_d(3) + Re(4) - \mu_r[R^*(3)] + X_R^{in}(4) - X_R^{out}(3) \\
&= \{R_e(0) - \mu_r[R^*(0)] \\
&\quad -R_d(1) - \mu_r[R^*(1)] \\
&\quad -R_d(2) + \gamma(1-\mu_n)N_d(0) - \mu_r[R^*(2)] - X_R^{out}(2)\} \\
&\quad -R_d(3) + \underline{Re(4)} - \mu_r[R^*(3)] + X_R^{in}(4) - X_R^{out}(3) \\
&= \{R_e(0) - \mu_r[R^*(0)] \\
&\quad -R_d(1) - \mu_r[R^*(1)] \\
&\quad -R_d(2) + \gamma(1-\mu_n)N_d(0) - \mu_r[R^*(2)] - X_R^{out}(2)\} \\
&\quad -R_d(3) + \underline{R_d(1) + \gamma(1-\mu_n)N_d(1)} - \mu_r[R^*(3)] + \underline{X_R^{in}(4)} - X_R^{out}(3) \\
&= \{R_e(0) - \mu_r[R^*(0)] \\
&\quad -R_d(1) - \mu_r[R^*(1)] \\
&\quad -R_d(2) + \gamma(1-\mu_n)N_d(0) - \mu_r[R^*(2) + \gamma N_d(2)] - X_R^{out}(2)\} \\
&\quad -R_d(3) + R_d(1) + \gamma N_d(1) - \mu_r[R^*(3)] + \underline{X_R^{out}(2)} - X_R^{out}(3) \\
&= R_e(0) - R_d(2) - R_d(3) + \gamma(1-\mu_n)[N_d(0) + N_d(1)] - X_R^{out}(3) \\
&\quad -\mu_r[R^*(0)] - \mu_r[R^*(1)] - \mu_r[R^*(2)] - \mu_r[R^*(3)] \\
R^*(4) &= R^*(3) + \gamma(1-\mu_n)N_d(3) - \mu_r[R^*(3)] \\
&= \{R^*(0) + \gamma(1-\mu_n)N_d(0) - \mu_r[R^*(0)] \\
&\quad +\gamma(1-\mu_n)N_d(1) - \mu_r[R^*(1)] \\
&\quad +\gamma(1-\mu_n)N_d(2) - \mu_r[R^*(2)] \\
&\quad +\gamma(1-\mu_n)N_d(3) - \mu_r[R^*(3)] \\
&= R_e(0) + \gamma(1-\mu_n)[N_d(0) + N_d(1) + N_d(2) + N_d(3)] \\
&\quad -\mu_r[R^*(0)] - \mu_r[R^*(1)] - \mu_r[R^*(2)] - \mu_r[R^*(3)]
\end{aligned}$$

<u>Stationary System.</u> If we set the immigration and emigration rates equal (i.e. $\mu_r = \mu_s = \mu_n = \kappa = C$), then verify that $Pop^*(t) = Pop^*(0) \; \forall \; t$.

Suppose:

$$\begin{aligned} R^*(0) &= R \\ S^*(0) &= S \\ N^*(0) &= N \end{aligned}$$

Then $Pop^*(0) = R + S + N$.

Consider $t = 1$, then we have the following when $\mu_r = \mu_s = \mu_n = \kappa = C$:

$$\begin{aligned} R^*(1) &= R^*(0) + \gamma(1-\mu_n)N_d(0) - \mu_r[R^*(0)] \\ &= (1-C)[R^*(0) + \gamma N_d(0)] \\ &= (1-C)[R + \gamma N_d(0)] \\ S^*(1) &= S^*(0) + (1-\gamma)(1-\mu_n)N_d(0) - \mu_s[S^*(0)] \\ &= (1-C)[S^*(0) + (1-\gamma)N_d(0)] \\ &= (1-C)[S + (1-\gamma)N_d(0)] \\ N^*(1) &= N^*(0) - N_d(0) + \kappa[Pop^*(0)] - \mu_n[N^*(0) - N_d(0)] \\ &= (1-C)[N^*(0) - N_d(0)] + C[Pop^*(0)] \\ &= (1-C)[N - N_d(0)] + C[R + S + N] \\ \Rightarrow Pop^*(1) &= R^*(1) + S^*(1) + N^*(1) \\ &= (1-C)[R + \gamma N_d(0) + S + (1-\gamma)N_d(0) + N - N_d(0)] + C[R + S + N] \\ &= R + S + N \\ &= Pop^*(0) \end{aligned}$$

For general $t$,

$$
\begin{aligned}
R^*(t+1) &= R^*(t) + \gamma(1-\mu_n)N_d(t) - \mu_r R^*(t) \\
S^*(t+1) &= S^*(t) + (1-\gamma)(1-\mu_n)N_d(t) - \mu_s S^*(t) \\
N^*(t+1) &= N^*(t) - N_d(t) + \kappa[R^*(t) + S^*(t) + N^*(t)] - \mu_n[N^*(t) - N_d(t)] \\
\Rightarrow Pop^*(t+1) &= R^*(t+1) + S^*(t+1) + N^*(t+1) \\
&= R^*(t) + S^*(t) + N^*(t) + C[Pop^*(t)] - C[Pop^*(t)] + (1-\mu_n)N_d(t) - (1-\mu_n)N_d(t) \\
&= Pop^*(t)
\end{aligned}
$$
